# Co-Occurrence of Two Plasmids Encoding Transferable *bla*_NDM-1_ and *tet*(Y) Genes in Carbapenem-Resistant *Acinetobacter bereziniae*

**DOI:** 10.3390/genes15091213

**Published:** 2024-09-17

**Authors:** Andrés Opazo-Capurro, Kyriaki Xanthopoulou, Rocío Arazo del Pino, Paulina González-Muñoz, Maximiliano Matus-Köhler, Luis Amsteins-Romero, Christian Jerez-Olate, Juan Carlos Hormazábal, Rodrigo Vera, Felipe Aguilera, Sebastián Fuller, Paul G. Higgins, Gerardo González-Rocha

**Affiliations:** 1 Laboratorio de Investigación en Agentes Antibacterianos (LIAA), Universidad de Concepción, Concepción 4070386, Chile; andopazo@udec.cl (A.O.-C.); paulinagonzalez@udec.cl (P.G.-M.); mmatus2016@udec.cl (M.M.-K.); lamsteins@udec.cl (L.A.-R.); christian.jerez@uss.cl (C.J.-O.); 2 Grupo de Estudio en Resistencia Antimicrobiana (GRAM), Universidad de Concepción, Concepción 4070386, Chile; 3 Institute for Medical Microbiology, Immunology and Hygiene, Faculty of Medicine and University Hospital Cologne, University of Cologne, D-50937 Cologne, Germany; kyriaki.xanthopoulou@uk-koeln.de (K.X.); rocio.arazo-del-pino@uk-koeln.de (R.A.d.P.); 4 German Centre for Infection Research (DZIF), Partner Site Bonn-Cologne, D-50937 Cologne, Germany; 5 Departamento de Ciencias Biológicas y Químicas, Facultad de Medicina y Ciencia, Universidad San Sebastián, Concepción 4070386, Chile; 6 Facultad de Odontología y Ciencias de la Rehabilitación, Universidad San Sebastián, Concepción 4070386, Chile; 7 Instituto de Salud Pública (ISP), Santiago 8320000, Chile; jchormazabal@ispch.cl; 8 Hospital de Urgencia Asistencia Pública, Santiago 8320000, Chile; rodrigo.verag@redsalud.gob.cl; 9 Departamento de Bioquímica y Biología Molecular, Facultad de Ciencias Biológicas, Universidad de Concepción, Concepción 4070386, Chile; faguilera@udec.cl (F.A.); sfuller2017@udec.cl (S.F.); 10 Centro de Biotecnología, Universidad de Concepción, Concepción 4070386, Chile; 11 Center for Molecular Medicine Cologne, University of Cologne, Faculty of Medicine and University Hospital Cologne, D-50937 Cologne, Germany

**Keywords:** *Acinetobacter bereziniae*, carbapenem-resistance, mobile-genetic elements, horizontal gene transfer

## Abstract

*Acinetobacter bereziniae* has emerged as a significant human pathogen, acquiring multiple antibiotic resistance genes, including carbapenemases. This study focuses on characterizing the plasmids harboring the *bla*_NDM-1_ and *tet*(Y) genes in two carbapenem-resistant *A. bereziniae* isolates (UCO-553 and UCO-554) obtained in Chile during the COVID-19 pandemic. **Methods**: Antibiotic susceptibility testing was conducted on UCO-553 and UCO-554. Both isolates underwent whole-genome sequencing to ascertain their sequence type (ST), core genome multilocus sequence-typing (cgMLST) profile, antibiotic resistance genes, plasmids, and mobile genetic elements. Conjugation experiments were performed for both isolates. **Results**: Both isolates exhibited broad resistance, including resistance to carbapenems, third-generation cephalosporins, fluoroquinolones, tetracycline, cotrimoxazole, and aminoglycosides. Both isolates belong to sequence type ST^PAS^1761, with a difference of 17 out of 2984 alleles. Each isolate carried a 47,274 bp plasmid with *bla*_NDM-1_ and *aph(3′)-VI* genes and two highly similar plasmids: a 35,184 bp plasmid with *tet*(Y), *sul2*, *aph(6)-Id*, and *aph(3″)-Ib* genes, and a 6078 bp plasmid containing the *ant(2″)-Ia* gene. Quinolone-resistance mutations were identified in the *gyrA* and *parC* genes of both isolates. Importantly, *bla*_NDM-1_ was located within a Tn*125* transposon, and *tet*(Y) was embedded in a Tn*5393* transposon. Conjugation experiments successfully transferred *bla*_NDM-1_ and *tet*(Y) into the *A. baumannii* ATCC 19606 strain, indicating the potential for horizontal gene transfer. **Conclusions**: This study highlights the critical role of plasmids in disseminating resistance genes in *A. bereziniae* and underscores the need for the continued genomic surveillance of this emerging pathogen. The findings emphasize the importance of monitoring *A. bereziniae* for its potential to cause difficult-to-treat infections and its capacity to spread resistance determinants against clinically significant antibiotics.

## 1. Introduction

Non-*baumannii Acinetobacter* species are increasingly prevalent in nosocomial infections and have been associated with antibiotic resistance genes that confer resistance to last-line antibiotics, such as carbapenems [1]. Among these, *A. bereziniae* (formerly designated as genomospecies 10), is considered as an emerging nosocomial pathogen involved in multiple infections, including urinary tract infections, pneumonia, chronic obstructive pulmonary disease, sepsis, and bacteraemia [2,3].

Carbapenems (i.e., imipenem and meropenem) are β-lactams antibiotics, considered the first line of treatment for infections against highly resistant Gram-negative isolates, including *Acinetobacter* species. These bactericidal antibiotics inhibit the bacterial cell-wall biosynthesis, producing the lysis of the bacterial cell [4].

Despite the important activity of these drugs, carbapenem resistance is becoming more frequent worldwide. In this sense, the World Health Organization (WHO) has deemed carbapenem-resistant Gram-negative bacteria as ‘critical priority’ pathogens for which the development of novel drugs is urgently needed [5].

The main mechanism of carbapenem resistance among Gram-negative organisms is mediated through enzymatic hydrolysis, i.e., carbapenemases [6]. In this regard, there are reports of carbapenem-resistant *A. bereziniae* strains associated with the production of diverse carbapenemases, including the metallo-β-lactamases (MBL) IMP-1, SIM-1, VIM-2, and NDM-1; and the overexpression of class D OXA-type carbapenemases including their intrinsic OXA-228-like [2,3,7,8].

Due to the above, polymyxins (i.e., colistin) and tetracyclines (including tetracycline, minocycline and tigecycline) are significant options to treat serious infections caused by carbapenem-resistant Gram-negative species, including *A. baumannii* [9,10]. Class B carbapenemases are metallo-β-lactamases (MBLs) and are clinically defined as one of the most relevant classes of carbapenemases [6].

The genes encoding these enzymes are frequently encoded in mobile genetic elements (MGEs) such as plasmids and/or transposons, facilitating their spread between different bacteria [6]. Among these enzymes, it has been documented that NDM-carbapenemases are associated with elevated mortality rates, posing a significant challenge to clinical management and a substantial risk to public health [11]. In Chile, NDM-enzymes have been emerging in hospital-acquired infections (HAIs) produced by Gram-negative species [12], denoting a significant challenge in the clinical setting.

On the other hand, tetracyclines are bacteriostatic broad-spectrum antibiotics that bind to the bacterial 30S ribosomal subunit and inhibit protein synthesis [13]. At present, there are three generations of tetracyclines available in clinical practice. The first generation is represented by tetracycline itself, whereas the second generation includes doxycycline and minocycline, and the third generation is represented by the glycylcycline tigecycline [14]. Significantly, an important role of tetracycline in the treatment of serious infections caused by carbapenem-resistant *Acinetobacter* spp. is its combined use with ampicillin/sulbactam [15].

Alarmingly, there are diverse reports of tetracycline resistance among Gram-negative pathogens, in which the main mechanism of resistance in *A. baumannii* corresponds to the activity of resistance/nodulation/cell division (RND) family-type efflux pumps, such as the Ade ATP-binding cassette (ABC) system [16]. AdeABC is also associated with elevated minimum inhibitory concentrations (MICs) for minocycline and tigecycline [17].

Furthermore, acquired efflux pumps that mediate resistance specifically to tetracyclines have been identified in *Acinetobacter* species, such as TetA and TetB, which belong to the major facilitator superfamily (MFS) [17]. Moreover, various *tet*(X) genes have been emerging, which encode for monooxygenases that can inactive all tetracyclines, including tigecycline [17]. Recently, a novel plasmid-encoded tetracycline resistance gene, *tet*(Y), that encodes for a tetracycline-specific MFS efflux pump [18], was identified in a clinical tigecycline-resistant *A. baumannii* isolate in China [19].

Ongoing evidence suggests that antibiotic resistance increased during the COVID-19 pandemic because of the over-use of antibiotics to treat seriously ill patients as a prophylaxis measure [20,21]. In 2021, the Instituto de Salud Publica of Chile (ISPCh) detected two isolates of NDM-producing *Acinetobacter* species from infections in hospitalized patients during the peak of the COVID-19 pandemic. This constituted the first detection of New Delhi metallo-β-lactamase (NDM)-positive *Acinetobacter* species in Chile. Alarmingly, these carbapenem-resistant isolates also exhibited resistance to multiple drugs, including tetracycline and aminoglycosides.

Consequently, the objective of this study was to characterize the genomic features and molecular epidemiology of these two carbapenem-resistant NDM-producing *Acinetobacter* spp. isolates recovered during the COVID-19 pandemic in Chile.

## 2. Materials and Methods

### 2.1. Bacterial Isolates

The *Acinetobacter* spp. isolates UCO-553 and UCO-554 were recovered from bronchial aspirate and blood samples, respectively. These samples were collected from two separate patients in a tertiary hospital in Santiago, Chile, during October 2020 (UCO-553) and February 2021 (UCO-554). Initially, both isolates were identified as members of the *Acinetobacter baumannii-calcoaceticus* complex. NDM production was confirmed using immunochromatographic tests (CORIS^®^, Gembloux, Belgium). The isolates were cultured in nutrient media and subsequently cryo-preserved in a mixture of culture broth and glycerol (50% *v*/*v*) at -80 °C for further analysis. For both isolates, we conducted a multiplex PCR for *Acinetobacter* species identification [22] and an additional multiplex PCR to detect various carbapenemase groups [23].

### 2.2. Antibiotic Susceptibility Tests (AST)

AST were carried out by disk diffusion according to the Clinical and Laboratory Standards Institute (CLSI) guidelines [24]. The antibiotics tested were imipenem (10 µg), meropenem (10 µg), cefotaxime (30 µg), ceftazidime (30 µg), cefepime (30 µg), ampicillin/sulbactam (10/10 µg), piperacillin/tazobactam (100/10 µg), gentamicin (10 µg), amikacin (30 µg), ciprofloxacin (5 µg), levofloxacin (5 µg), tetracycline (30 µg), and sulfamethoxazole/trimethoprim (1.25/2375 µg). Furthermore, the colistin minimum inhibitory concentration (MIC) was determined by broth microdilution using the ComASP colistin kit (Liofilchem^®^, Teramo, Italy), and the tetracycline, minocycline and tigecycline MICs were determined utilizing the SensiTitre MIC system (ThermoFisher^®^, Waltham, Massachusetts, USA). The tigecycline and colistin MIC values were interpreted according to Seifert et al. [25] and CLSI [24], respectively.

### 2.3. Whole-Genome Sequencing (WGS) and In Silico Analyses

Genomic DNA (gDNA) from both isolates was extracted using the InstaGene matrix (Bio-Rad^®^, Hercules, CA, USA) following the manufacturer’s protocol. Whole-genome sequencing (WGS) was conducted using both short- and long-read sequencing techniques. Initially, libraries were prepared with the Illumina^®^ DNA Prep kit and sequenced on an Illumina NextSeq 2000, generating 2 × 150 bp reads. Demultiplexing, quality control, and adapter trimming were performed using bcl-convert (v3.9.3). For long-read sequencing, gDNA was extracted using the Wizard^®^ HMW DNA Extraction Kit (Promega, Madison, USA) and sequenced with Oxford Nanopore Technologies (ONT). Library preparation, MinION sequencing, and bioinformatic processing were conducted as previously described [26]. The combined long- and short-reads were assembled using the Unicycler assembly pipeline [27].

Species determination was conducted by comparing the genomes against the GenomesDB database hosted on the JSpeciesWS server [28]. In silico detection of antibiotic-resistance genes (ARGs) and sequence typing (ST) was performed using ABRIcate with ResFinder v4.4.2 and MLST v2.22.0, respectively, integrated into the Galaxy platform [29]. Plasmid typing was carried out by the MOB-typer tool [30]. Plasmids were annotated using the NCBI Prokaryotic Genome Annotation Pipeline (PGAP), and gene cluster comparisons were built using Clinker (v0.0.23) [31] after the manual curation of the sequences using the Artemis software v18.2.0. The Proksee tool (https://proksee.ca, accessed on January 2024) was utilized for analyzing and visualizing the sequenced genomes of both isolates [32].

### 2.4. Core-Genome MLST (cgMLST)

To investigate the molecular epidemiology of the isolates, an ad hoc core genome multi-locus sequence typing (cgMLST) scheme was developed using the cgMLST target definer function of the Ridom SeqSphere+ software (Ridom GmbH, Münster, Germany) with default parameters. The *A. bereziniae* strain XH901 (NZ_CP018259.1) served as the reference genome, and the cgMLST scheme comprised a core genome of 2984 alleles.

### 2.5. Conjugation Experiments

Mating experiments were performed to evaluate the transmissibility of the *bla*_NDM-1_ and *tet*(Y) genes from *A. bereziniae* UCO-553 and UCO-554 strains (imipenem-resistant, tetracycline-resistant, and chloramphenicol-susceptible) to *A. baumannii* ATCC 19,606 (imipenem-susceptible, tetracycline-susceptible, and chloramphenicol-susceptible). Isolates were incubated overnight at 37 °C in lysogeny broth (LB) and subsequently mixed at a 1:10 donor/recipient ratio. The mixtures were incubated for 36 h at 37 °C without shaking, and transconjugant cells were selected on LB plates supplemented with 16 µg/mL of tetracycline and 128 µg/mL of chloramphenicol. Conjugation was verified by PCR, which assessed species identification and the presence of *bla*_NDM-1_ and *tet*(Y) genes in the transconjugant bacteria.

## 3. Results

The initial multiplex PCR analyses confirmed that both isolates were NDM-producing *Acinetobacter* species, distinct from *A. baumannii*. Additionally, disk diffusion assay results (Table 1) indicated that both isolates exhibited resistance to carbapenems (imipenem and meropenem), cephalosporins (cefepime, ceftazidime, and cefotaxime), β-lactam/β-lactamase inhibitor combinations (ampicillin/sulbactam and piperacillin/tazobactam), fluoroquinolones (ciprofloxacin and levofloxacin), aminoglycosides (gentamicin and amikacin), tetracycline, and sulfamethoxazole/trimethoprim. They demonstrated intermediate resistance to colistin (MIC ≤ 0.25 µg/mL). Conversely, both isolates were susceptible to minocycline and tigecycline (MIC ≤ 1 µg/mL). Consequently, UCO-553 and UCO-554 were classified as extensively drug-resistant (XDR) [33].

The genome sizes of UCO-553 and UCO-554 are highly similar, comprising 4,557,265 bp, and 4,557,136 bp, respectively. Both strains have a GC content of 38.07%. Moreover, each isolate possesses four plasmids of the same size, ranging from 5594 bp to 47,274 bp. The species identification was confirmed using the genome sequences in the tetra-correlation comparison from the JSpeciesWS server. Likewise, they belonged to the ST^PAS^1761, and cgMLST revealed that they differ in 17/2984 alleles, which is interpreted as not originating from the same source. We determined this by using the cut-off for transmissions for *A. baumannii*, which was determined to be up to eight alleles different using cgMLST [34].

Regarding antibiotic-resistance gene content, UCO-553 and UCO-554 harbor the chromosomally encoded *bla*_OXA-931_ that belongs to the intrinsic *bla*_229_-like group [35]. There was no insertion sequence (IS) either upstream or downstream of *bla*_OXA-931_. In addition to the intrinsic OXA-type β-lactamase, the acquired resistome of both isolates was the same, constituted by the aminoglycoside resistance genes *aph(3′)-VI*, *aph(6′)-Id* (*strB*), *aph(3″)-Ib* (*strA*), and *ant(2″)-Ia*, the sulphonamide resistance gene *sul2*, the carbapenemase gene *bla*_NDM-1_, and *tet*(Y) that mediates resistance to tetracycline [36]. Moreover, isolates UCO-553 and UCO-554 exhibit mutations at different positions, specifically Met125Ile in GyrA and Phe84Tyr in ParC, with the *A. bereziniae* strain GD03185 genome (GenBank accession number CP066119.1) serving as the reference.

**Figure 1:**
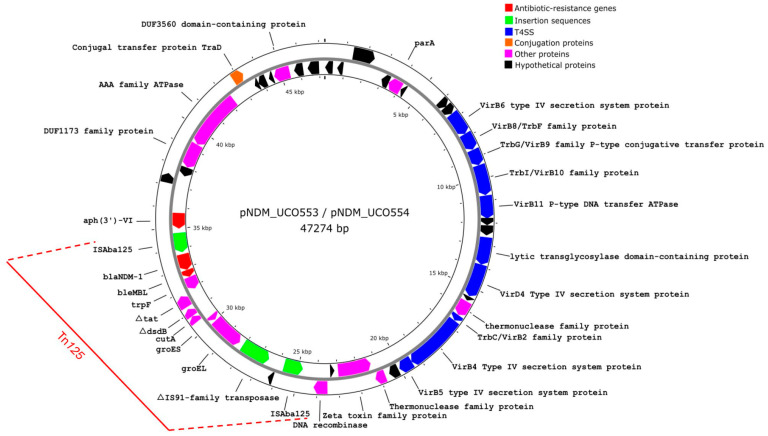
Graphical representation of 47,274 bp plasmids harbored by *A. bereziniae* UCO-553 and UCO-554 strains. Arrows indicate the length and directions of genes and ORFs. Tn*125* is indicated in the red line. Genes annotations were performed by the NCBI Prokaryotic Genome Annotation Pipeline (PGAP), whereas the plasmid was visualized using Proksee and edited using Inkscape v1.2.

Furthermore, we identified the presence of identical 47,274 bp plasmids harboring the *bla*_NDM-1_ gene in both isolates (pNDM_UCO553 and pNDM_UCO554, accession numbers CP133667.1 and CP123919, respectively), exhibiting 100% coverage and 100% identity (Supplementary Materials), and belonging to the MOBq-type (Figure 1). A BLAST analysis revealed that these plasmids are highly similar to a plasmid previously reported in *A. baumannii* from Colombia in 2016, with 100% coverage and 99.99% identity (accession number CP010399), which also carries the *bla*_NDM-1_ and *aph(3′)-VI* resistance genes. Furthermore, the *bla*_NDM-1_ gene in both UCO-553 and UCO-554 is located within a Tn*125* transposon (Figure 1), a feature also present in the Colombian *A. baumannii* strain. As shown in Figure 1, this plasmid also contains genes encoding for a type IV secretion system (T4SS) proteins, which are protein involved in the translocation of proteins and/or DNA [37].

Commonly, Tn*125* encodes for the *bla*_NDM-1_ in Gram-negative species [38]. Specifically, this element is delimited by two IS*Aba125* copies, forming a composite transposon that is able to be mobilized, facilitating the spread of NDM-1 [39].

**Figure 2:**
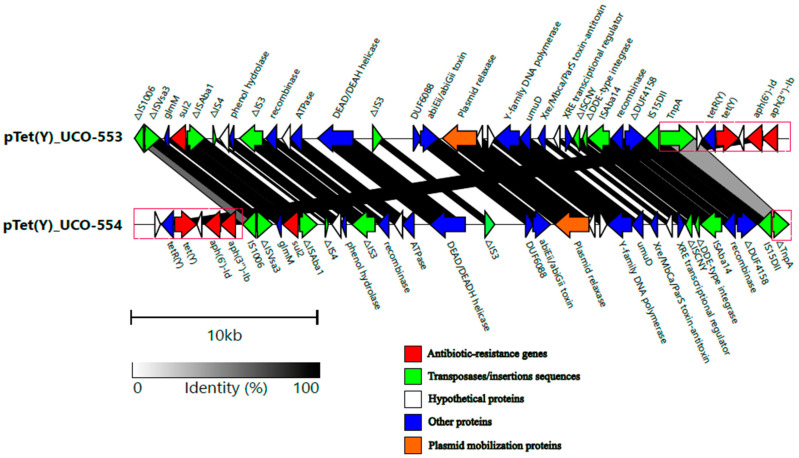
Comparative linear maps of the *tet*(Y)-encoding plasmids harbored by UCO-553 and UCO-554 strains. Arrows indicate the length and directions of genes and ORFs. Antibiotics-resistant genes (ARGs) are marked in red, insertions sequences (IS) and transposases are in green, plasmid mobilization proteins genes in yellow, other genes are in blue and hypothetical proteins in white. Tn*5393* is denoted in the red rectangle. Gene annotations were performed by the NCBI Prokaryotic Genome Annotation Pipeline (PGAP), whereas genomic alignment was facilitated by clinker v0.0.23 [31] and the figure was processed using Inkscape v1.2.

Additionally, both isolates contained highly similar MOBp-type plasmids of 35,184 kb (100% coverage and 99.99% identity) that harbor the *tet*(Y) gene in addition with *sul2, aph(3″)-Ib (strA)* and *aph(6′)-Id (strB)* and were classified as conjugative [40] (Figure 2). According to the MOB-typing results, the closest sequence (39% coverage) corresponds to a plasmid identified in *A. pittii* in Taiwan (accession number CP033537), which does not harbor the *tet*(Y) gene. Interestingly, the *tet*(Y), *aph(6)-Id* and *aph(3″)-Ib* genes were located in a Tn*5393*-like transposon (Figure 2). Although both plasmids are similar, the *tnpA* gene of Tn*5393* was intact in UCO-553, whereas this gene was incomplete in UCO-554 (Figure 2). Furthermore, *bla*_NDM-1_- and *tet*(Y)-containing plasmids were mobilized by conjugation, resulting in the increase in the MIC for imipenem (x > 340) and tetracycline (x 256), whereas the MIC for tigecycline did not change (Table 2). These results demonstrate that both plasmids are mobilizable.

## 4. Discussion

*A. bereziniae* has been emerging as a relevant human pathogen due to its ability to develop resistance to several antibiotics, including carbapenems [41]. Here, we described the genomic features of two *A. bereziniae* isolates recovered from human infections during the COVID-19 pandemic in Chile.

These isolates displayed a broad resistance phenotype, including resistance to β-lactams, such as carbapenems, tetracycline, aminoglycosides, and sulfamethoxazole/trimethoprim. In this context, minocycline, tigecycline and colistin were active against the isolates; thus, the therapeutic options were limited.

We determined that both isolates were resistant to fluoroquinolones. Interestingly, it has been demonstrated that fluoroquinolone resistance is commonly mediated by the Ser83Leu substitution in GyrA and the Ser80Leu substitution in ParC within the *A. baumannii-calcoaceticus* complex [42].

However, the isolates UCO-553 and UCO-554 exhibit mutations at different positions, specifically Met125Ile in GyrA and Phe84Tyr in ParC, with the *A. bereziniae* strain GD03185 genome (GenBank accession number CP066119.1) serving as the reference. These substitutions differ from those typically associated with fluoroquinolone resistance in *A. baumannii*. Therefore, further investigation is required to understand the role of these novel substitutions in fluoroquinolone resistance in *A. bereziniae* [42].

Furthermore, both isolates contained two main plasmids harboring genes that mediate resistance to relevant antibiotics, such as *bla*_NDM-1_ and *tet*(Y). Relevantly, these genes were transferred by conjugation, highlighting their potential to be successfully disseminated among clinically relevant bacteria. Significantly, these genes were contained in transposons, which may help in their dissemination.

We found the Tn*125* transposon carrying the *bla*_NDM-1_ gene in identical plasmids of 47,274 bp in both isolates. This transposon is frequently associated with the *bla*_NDM-1_ gene and is significant in its dissemination [11], playing an important role in the dissemination of *bla*_NDM_.

Previously, Mo et al., have detected the *bla*_NDM-1_ gene in three *A. bereziniae* isolates collected in two major tertiary hospitals in China, in which this carbapenemase gene was also located in a Tn*125* transposon [43]. Furthermore, since the first detection of *A. bereziniae* carrying the *bla*_NDM-1_ in Brazil in 2014 [44], other cases in Argentina [45,46], Japan [47], Australia [48], and China [7,43] have been documented.

The impact of NDM-carbapenemase carriage on patient outcomes has been previously established. For instance, Pudpong et al. found that mortality rates for infections caused by Enterobacterales harboring NDM-1 or NDM-1/OXA-48 were higher than those associated with OXA-48 carbapenemase alone [49]. Consequently, the spread of plasmids encoding NDM-1 carbapenemase may contribute to increased mortality in these infections, highlighting the significant influence of these mobile genetic elements on patient outcomes. Furthermore, we identified the *aph(3′)-VI* gene in both plasmids conferring resistance to gentamicin and amikacin, similar to the findings in the *A. baumannii* isolate from Colombia.

Moreover, we determined that these plasmids also contain genes encoding a type IV secretion system (T4SS) [37]. In this sense, the virB proteins that conforms the T4SS have been associated with the plasmid mobilization [49]. Interestingly, the *virB* genes detected in the 47,274 bp plasmids (Figure 1) have been associated with conjugative type IV secretion systems, in which diverse components, such as channel formation and pore structure (virB8 and virB6), pore structure/substrate transfer (virB9 and virB10), ATP-hydrolysis (VirB11), ATPase (VirB4), adhesion (virB5), major pilus subunit (VirB2), and DNA binding (VirD4), are involved in the conjugation process, facilitating the dissemination of these plasmids.

On the other hand, both isolates contained plasmids of 35,184 bp containing the *tet*(Y) gene embedded in a Tn*9353* transposon. To the author’s knowledge, this is the first description of the tetracycline resistance gene *tet*(Y) in *A. bereziniae* isolates. This gene was originally identified in a clinical *A. baumannii* strain in China recovered from a bronchoalveolar lavage sample in 2016 [19]. In that report, *tet*(Y) was also located in a Tn*5393* transposon embedded in a 72,126 bp plasmid that was not typeable [19].

As illustrated in Figure 2, both 35,184 bp plasmids possess a variety of toxin–antitoxin (TA) systems. Specifically, they harbored the abiEii/abiGii toxin–antitoxin system, which is part of the abortive infection (Abi) mechanism [50]. Moreover, these plasmids contain an additional TA system, Xre/Mbca/ParS (Figure 2), which facilitates the stabilization of these plasmids. Moreover, the presence of genes involved in plasmid stabilization (TA systems), conjugation (T4SS), and a myriad of IS (i.e., IS*Aba215*) and transposons (i.e., Tn*125* and Tn*5393*), could explain their success at dissemination of relevant ARGs, such as *bla*_NDM-1_ and *tet*(Y).

Although we determined that *tet*(Y) was inserted in the same transposon compared with the report of Wang et al., the plasmids are different. Interestingly, the Tn*5393* transposon was first identified on a plasmid from *Klebsiella pneumoniae*, demonstrating its ability to be transferred at the interspecies level, thus contributing to its mobilization and dissemination [51].

Remarkably, Kyselková et al. [36] identified the *tet*(Y) gene in plasmids recovered from soil and manure samples, in which *tet*(Y)*-tet*(R), in addition to the *aph(6′)-Id* and *aph(3′′)-Ib* genes, was located in a structure similar to Tn*5393*, highlighting the importance of this transposon in the mobilization of these genes. In this sense, fragments of Tn*5393* appear to be more common than the intact transposon in plasmids [51], which is concordant with our findings.

Recently, Stehling et al. described an *A. bereziniae* isolate collected from an infected semiaquatic turtle that contains the novel Tn*9393m* that is associated with the *qnrB19* gene that confers resistance to fluoroquinolones [41]. This finding underscores the relevance of this transposon in the carriage and dissemination of ARGs, which is concordant with our findings. However, this study included a comparative genomic analysis of the *A. bereziniae* genomes available in public databases, from which the authors concluded that most of the isolates did not carry any ARGs [41]. Moreover, the authors determined that some strains from Asia and Brazil harbored six or more antibiotic resistance genes [41]; therefore, our results contribute to the knowledge of *A. bereziniae* as an important resistant pathogen, as we identified eight antibiotic resistance genes in each isolate, demonstrating that this species could accumulate and disseminate ARGs.

Furthermore, while the ARG content of both isolates aligns with their observed resistance phenotypes, there is a discrepancy concerning their susceptibility to minocycline and tigecycline.

Despite the above, our study demonstrated that both isolates were susceptible to these drugs despite the presence of the *tet*(Y) gene, which encodes for an MFS efflux-pump that has been previously associated with minocycline- and tigecycline resistance in *A. baumannii* [18,19]. In this sense, in the same publication, the authors demonstrated that the mobilization of *tet*(Y) into a susceptible strain resulted in a 4-fold increase in tigecycline MIC [19], and when this gene was co-transferred with *tet*(A), the tigecycline MIC showed a 128-fold increase.

However, our results showed that UCO-553 and UCO-554 were susceptible to minocycline and tigecycline (MIC ≤ 1 µg/mL), indicating that the sole presence of Tet(Y) does not confer resistance to second- and third-generation tetracyclines in *A. bereziniae*. These results highlight the relevance of highly resistant emerging species causing infections in the clinical setting. Specifically, as both *A. bereziniae* strains included in this study were resistant to last-line antibiotics, including carbapenems, the remaining therapeutic options are colistin and tigecycline; hence, it is important to be aware of the presence of these types of strains in order to adopt a effective therapy.

In conclusion, our study characterized the first two NDM- and Tet(Y)-producing *A. bereziniae* isolates collected in Chile that are resistant to multiple antibiotics, including carbapenems and tetracycline. As most of the resistance genes are plasmid-encoded, this underscores the importance of mobile genetic elements in the dissemination of resistance genes among clinical isolates of emerging species that could act as a reservoir of these genes. Finally, our findings emphasize the significance of conducting genomic surveillance to detect highly resistant strains that pose a serious threat to public health.

## Figures and Tables

**Table 1:** Susceptibility profiles of *A. bereziniae* UCO-553 and *A. bereziniae* UCO-554 strains included in this study.

Strains	Disk Diffusion Test													MIC (µg/mL)		
	IMP	MEM	FEP	CAZ	CTX	SAM	TZP	CIP	LEV	GEN	AMK	TET	SXT	CST	MIN	TGC
UCO-553	R	R	R	R	R	R	R	R	R	R	R	R	R	≤0.25 (I)	≤1 (S)	≤1 (S)
UCO-554	R	R	R	R	R	R	R	R	R	R	R	R	R	≤0.25 (I)	≤1 (S)	≤1 (S)

IMP: imipenem; MEM: meropenem; CAZ: ceftazidime; CTX: cefotaxime; TZP: piperacillin/tazobactam; CIP: ciprofloxacin; LEV: levofloxacin; GEN: gentamicin; AMK: amikacin; TET: tetracycline; SXT: sulfamethoxazole/trimethoprim; CST: colistin; MIN: minocycline; TGC: tigecycline. MIC: minimum inhibitory concentration. R: resistant; I: intermediate; S: susceptible.

**Table 2:** Minimum inhibitory concentrations (MICs) of the antibiotics used in the conjugation assay.

Strains	CHL (µg/mL)	IMP (µg/mL)	TET (µg/mL)	MIN (µg/mL)	TGC (µg/mL)
*A. bereziniae* UCO-553	8	>32	256	≤1	≤1
*A. bereziniae* UCO-554	4	>32	256	≤1	≤1
*A. baumannii* ATCC 19606	128	0.094	1	≤1	≤1
*A. baumannii* Tc-553	128	>32	256	≤1	≤1
*A. baumannii* Tc-554	128	>32	256	≤1	≤1

CHL: chloramphenicol; IMP: imipenem; TET: tetracycline; MIN: minocycline; TGC: tigecycline; Tc: transconjugant.

## Data Availability

This Whole Genome Shotgun project has been deposited at DDBJ/ENA/GenBank under the accession numbers CP133666.1 (UCO-553 chromosome), CP133667 to CP133670 (UCO-553 plasmids), and CP123918 (UCO-554 chromosome), CP123919 to CP123922 (UCO-554 plasmids).

## Supplementary Materials

The following supporting information can be downloaded at: https://www.mdpi.com/article/10.3390/genes15091213/s1. Figure S1: coverage and identity of blaNDM-1 gene.
