# Supplementary figures and images for: Co-Occurrence of Two Plasmids Encoding Transferable blaNDM-1 and tet(Y) Genes in Carbapenem-Resistant Acinetobacter bereziniae
Source: Genes (Basel). 2024 Sep 17;15(9):1213. doi: 10.3390/genes15091213 (PMC11431271; doi:10.3390/genes15091213)

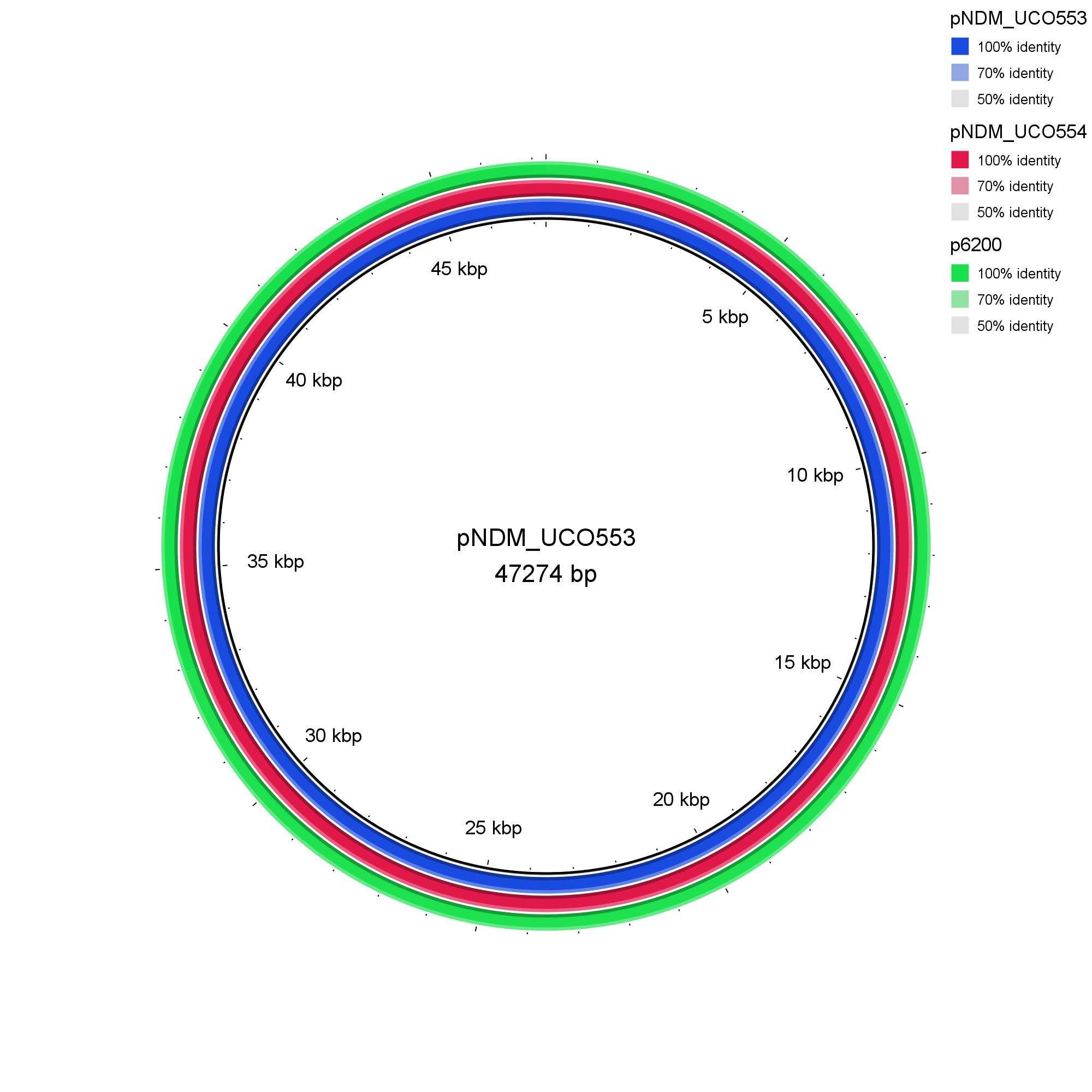

Supplement: Supplementary file 1 [file genes-15-01213-s001.zip › genes-3196360-supplementary.jpg]
